# Research Trends of Biodegradation of Cooking Oil in Antarctica from 2001 to 2021: A Bibliometric Analysis Based on the Scopus Database

**DOI:** 10.3390/ijerph18042050

**Published:** 2021-02-19

**Authors:** Khadijah Nabilah Mohd Zahri, Azham Zulkharnain, Suriana Sabri, Claudio Gomez-Fuentes, Siti Aqlima Ahmad

**Affiliations:** 1 Department of Biochemistry, Faculty of Biotechnology and Biomolecular Sciences, Universiti Putra Malaysia, Serdang 43400 UPM, Selangor, Malaysia; khadijahnabilah95@gmail.com; 2 Department of Bioscience and Engineering, College of Systems Engineering and Science, Shibaura Institute of Technology, 307 Fukasaku, Minuma-ku, Saitama 337-8570, Japan; azham@shibaura-it.ac.jp; 3 Department of Microbiology, Faculty of Biotechnology and Biomolecular Sciences, Universiti Putra Malaysia, Serdang 43400 UPM, Selangor, Malaysia; suriana@upm.edu.my; 4 Department of Chemical Engineering, Universidad de Magallanes, Avda. Bulnes, Punta Arenas 01855, Chile; claudio.gomez@umag.cl; 5 Center for Research and Antarctic Environmental Monitoring (CIMAA), Universidad de Magallanes, Avda. Bulnes, Punta Arenas 01855, Chile; 6 National Antarctic Research Centre, B303 Level 3, Block B, IPS Building, Universiti Malaya, Kuala Lumpur 50603, Malaysia

**Keywords:** biodegradation, bioremediation, cooking oil, Antarctic

## Abstract

In the present age, environmental pollution is multiplying due to various anthropogenic activities. Pollution from waste cooking oil is one of the main issues facing the current human population. Scientists and researchers are seriously concerned about the oils released from various activities, including the blockage of the urban drainage system and odor issues. In addition, cooking oil is known to be harmful and may have a carcinogenic effect. It was found that current research studies and publications are growing on these topics due to environmental problems. A bibliometric analysis of studies published from 2001 to 2021 on cooking oil degradation was carried out using the Scopus database. Primarily, this analysis identified the reliability of the topic for the present-day and explored the past and present progresses of publications on various aspects, including the contributing countries, journals and keywords co-occurrence. The links and interactions between the selected subjects (journals and keywords) were further visualised using the VOSviewer software. The analysis showed that the productivity of the publications is still developing, with the most contributing country being the United States, followed by China and India with 635, 359 and 320 publications, respectively. From a total of 1915 publications, 85 publications were published in the *Journal of Agricultural and Food Chemistry*. Meanwhile, the second and third of the most influential journals were *Bioresource Technology* and *Industrial Crops and Products* with 76 and 70 total publications, respectively. Most importantly, the co-occurrence of the author’s keywords revealed “biodegradation”, “bioremediation”, “vegetable oil” and “Antarctic” as the popular topics in this study area, especially from 2011 to 2015. In conclusion, this bibliometric analysis on the degradation of cooking oil may serve as guide for future avenues of research in this area of research.

## 1. Introduction

The world has become contaminated with toxic pollutants from multiple sources due to increasing human activities. Amongst the most prominent pollutants released from anthropogenic activities are petroleum hydrocarbons and polycyclic aromatic hydrocarbons (PAHs), which include cooking oil as it is also categorised as a hydrocarbon compound [1,2]. Waste cooking oil is the major waste generated from food processing industries, dairy industries, kitchen activities, bakeries and beverage industries [3]. This problem is also one of the main concerns of environmentalists as it is discharged into drains, running water or sewages without prior treatment, which causes damage to the environment. This shows that hydrocarbon pollution is a global problem that has attracted the attention of many countries and researchers. This includes the Antarctic continent, the only continent on Earth without indigenous human inhabitants [4]. However, it is almost impossible to avoid some waste oil from entering the Antarctic environment from stations and ships, while the risk of spillage events remains during storage and transport of fats.

Waste cooking oil can be harmful to the microorganisms and other organisms. The presence of toxic, organic and volatile compounds such as acrylamide, aldehyde, 4-hydroxymethylfurfural in heated cooking oil (waste oil) has been known to have mutagenic and carcinogenic activities [5,6]. Additionally, toxic compounds in the oil can be readily dissolved into the water and absorbed into living cells, eventually killing plants and animals. Furthermore, the consumption of heated cooking oil or the produced toxic compounds can cause inflammation, endothelial dysfunction, high blood pressure and neurodegenerative diseases [7]. As such, this topic is connected to other fields such as pharmacology, toxicology, medicine and chemistry in addition to bioremediation research.

Bioremediation plays a significant role in cleaning the environment. It is more reliable and more eco-friendly, which utilises microbes and plants’ natural ability to remove or neutralise the pollutants present in the environment [8,9,10]. Microorganisms are crucial in regulating the biogeochemical cycle and preserving the Earth, but they can also clean the environment from contaminants [9,11]. Nevertheless, long period is required for an effective bioremediation, and in the case of greatly polluted environment, the process is less effective usually due to the limited of abiotic factors such as nutrient availability, oxygen concentration and temperature [12]. Thus, the applications of bioremediation should be developed and explored more to generate significant data in this field.

Bibliometric methods mainly involve various statistical methods of bibliography analysis to evaluate and measure the literature’s progress in a particular field area [13]. Generally, the bibliometric method is used to analyse current research and development trends in a selected field based on co-occurrence, co-citation, co-author, co-word and bibliographic coupling. Nevertheless, in this study, the bibliometric analysis is primarily aimed to accomplish the following objectives: (1) to determine the productivity and reliability of this research topic on remediation of cooking oil for present-day, (2) to explore the past and present progresses of articles published and global trends in the study of cooking oil degradation, (3) to identify the most contributing countries and journals on the topic of concern, (4) to reveal the most and least of research studies according to the author’s keywords, (5) to show the dispersion and intersection of researches on the topic of concern to other different subject areas.

## 2. Theoretical Framework/Background

We decided to focus on polar research, where hydrocarbons pollution in the Antarctic has occurred due to the fuel oil that has been widely used there as a source of energy [14]. The majority of Antarctic research stations are located on ice-free coastal areas to allow a more accessible station construction and re-supply by ship. Terrestrial oil spills tend to be found at sites of human activity. Although the risk of cooking oil pollutants to the Antarctic environment is minor, researchers should be prepared with all the possibilities that might happened in the Antarctic, such cooking oil spills could occur during the loading and unloading of the waste cooking oil from the Antarctica to the outside of continents for waste disposal. More than 50 stations and bases in the Antarctica are still currently active for research purposes with military forces from many countries providing support to them [15]. Since cooking oil is used for food preparation in the kitchen for researchers and military forces, there exist the real possibility of domestic oil spillage.

It is useful to have an overview of the Antarctic environment to understand the challenges of carrying out bioremediation there. The average yearly temperature ranges from about −10 °C (Antarctic coast) to −60 °C (highest parts). Nearby the coastline, the temperature can exceed +10 °C (summer) and −40 °C (winter). Over the centre of the continent, the temperature is colder, which is about −30 °C (summer) to −80 °C (winter) [16]. This climate condition is one of the limitations in the remediation of oil in the Antarctic. Low ambient temperatures result in increased viscosity of oil, decreased evaporation of volatiles and increased water solubility, thus delaying the remediation processes [17].

Numerous remediation techniques have been developed during the past few decades on different aspects other than biological methods, such as physiochemical methods [18]. In Antarctica, the oil spills was cleaned through the absorption process using the absorbent pad [14]. The past method in the Antarctic mostly involved in the excavation process to the waste disposal site, followed by monitoring and evaluation. However, there has been a little report on the ecological impacts of waste disposal sites and the outcomes of the remediation attempts [19]. Meanwhile, for the water treatment plant consisted of two stages separation to remove solids and dissolved contaminants, which comprised ferric chloride coagulant to induce flocculation. The section for the removal of dissolved metal contaminants incorporated two ion-exchange columns operating series and the sample were collected from the outlet of the column to assess the performance of the water treatment. Other than that, alternative physiochemical remediation technologies such as permeable reactive barriers, vapour extraction and chemical oxidation were applied in Antarctica [20]. Although all the remediation techniques that have been discussed previously was used specifically in diesel or petroleum oil remediation, these techniques also can be applied for cooking oil since they share same physical properties, such as high viscosity and low density compared to water.

## 3. Bibliometric Analysis

The bibliometric analysis focused on the past 20 years (2001 to 2021) of available publications on the degradation of cooking oil or vegetable oil.

### 3.1. Data Extraction

The source of data was the Scopus database. The years from 2001 until 2021 were selected and the main limits searched included the article title, abstract and keywords. The search words used in this study were “bioremediation” or “biodegradation” or “remediation” or “degradation” and “vegetable oil” OR “cooking oil” or “Antarctic”, which exhibited an output of 3567 total publications. There were no single articles and publications that were excluded during data extraction for this analysis as it can cause bias to other studies [21].

### 3.2. Contribution of Countries

**Figure 1:**
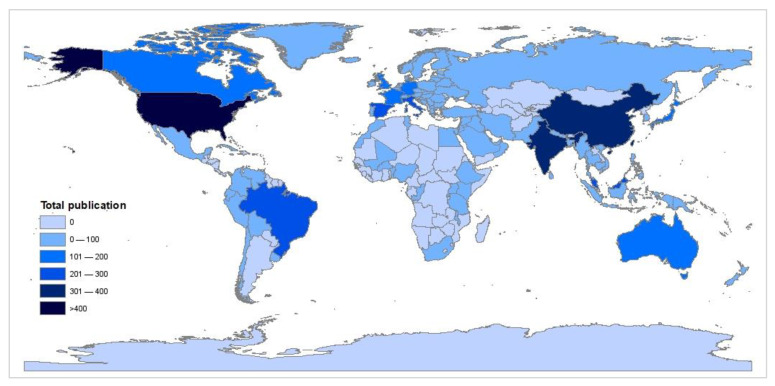
Global national map on the distribution of publication.

The number of publications in every field is the key to assessing global trends and determining the reliability of the research topic for the current study. The bibliometric analysis of the selected research topic revealed that out of 105 countries, the United States, China, India, Spain, Italy, Brazil, Malaysia, Canada, United Kingdom and Japan, are the top 10 countries that contribute the most in terms of publication. Hong Kong, Taiwan and Macau were included in mainland China. Figure 1 shows a world map with different colours indicating the distribution of the publications on this topic in each country, with darker colours indicating more publications. The United States leads the world in the highest number of publications on remediation of cooking oil, with 635 total publications for 20 years.

**Figure 2:**
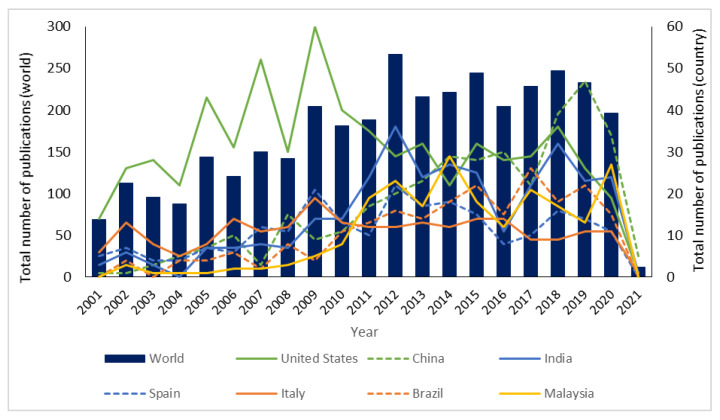
Distribution of global yearly publications on cooking oil remediation of selected countries.

Looking at the general trend, the total number of publications on this research topic increased from within the last two decades (Figure 2), though number fluctuated by year. Seven countries that published more than 200 publications were further analysed in each year. Although the United States was the highest contributing country, their interest in this study declined over the past 10 years. Nevertheless, other countries, particularly China, India and Brazil continues to publish high numbers of papers in this research topic. China’s and India’s cooking oil remediation research has evolved significantly from 2016 to 2018. As stated by Panadare and Rathod, China and India which are countries with very large populations, produced large amounts of waste cooking oil (about 4.5 million and 0.167 million tons per year, respectively) [22].

### 3.3. Journal Performance 

The most published journals in this research topic can be identified through bibliometric studies and cross referenced with their impact factors. Overall, 154 different journals were identified from the database in the remediation of cooking oil. Table 1 shows the journals that published in this subject area, with *The Journal of Agriculture and Food Chemistry* having the highest total percentage of publications (4.44%), followed by *Bioresource Technology* (3.97%) and *Industrial Crops and Products* (3.66%). As mentioned by ACS Publications in 2020, *The Journal of Agriculture and Food Chemistry* encourages research on chemistry or biochemistry as a major component combined with biological, toxicological, sensory or nutritional evaluation related to agriculture or food [23]. The wide range of subjects provided by this journal allowed many researchers to publish in this journal and eventually led to many publications. Yet, the journal with the highest impact factor among the top 20 productive journals, which is the *The Journal of Hazardous Materials*, was only ranked 9th, with an impact factor of 9.038 according to the InCites Journal Citation Reports.

**Figure 3:**
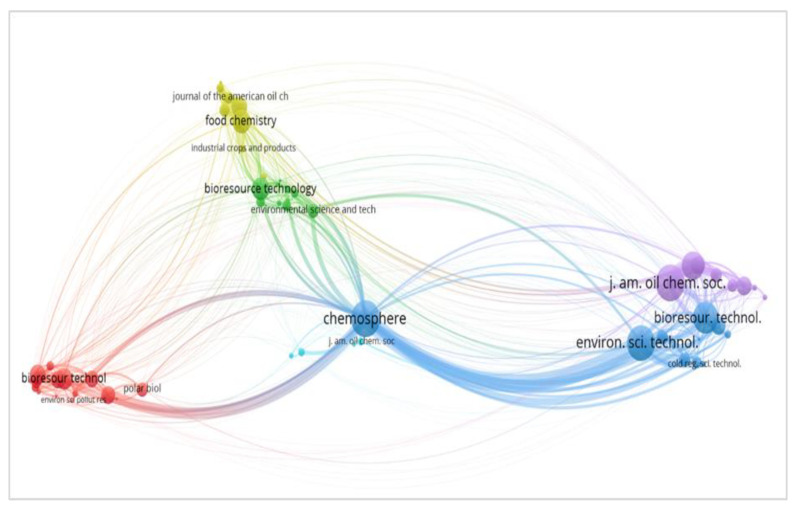
Bibliometric map based on co-citation analysis with network visualization.

Several journals have both high citations and high impact factors. However, the most cited among the top 20 productive journals for the topic of cooking oil remediation was the *Chemosphere Journal* with a total of 1236 co-citations and an impact factor of 5.778. This can also be understood based on the co-citation bibliometric map shown in Figure 3, which was analysed using VOSviewer Version 1.6.15 (Leiden University, Leiden and The Hague, South Holland, Netherlands). According to Abbas et al. the strength of connections between journals depends on the distance of the link. The closer the link between subjects, the bigger the connection [24]. The total link strength of *Chemosphere Journal* with other journals was 13,433 and is closely related to 10 other journals (*Applied Microbiology and Biotechnology, Bioresource Technology, Cold Regions Science and Technology, Environmental Science and Pollution Research, Environmental Science and Technology, International Biodeterioration and Biodegradation, Journal of Environmental Management, Journal of Hazardous Materials* and *Polar Biology* as well as *Science of the Total Environment*).

### 3.4. Contribution of Various Fields

**Figure 4:**
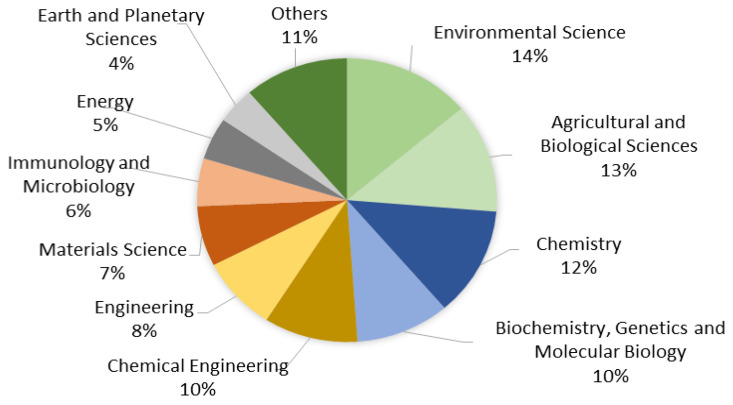
Distribution of fields that contributed to publications on cooking oil remediation.

We studied the contribution of various fields to identify the evolutionary trends and emerging research hotspots related to cooking oil remediation [25]. Figure 4 illustrates the distribution of fields, where environmental science contributed the highest percentage of publications (14%), followed by agricultural and biological sciences (13%), chemistry (12%), biochemistry, genetics and molecular biology (10%) as well as chemical engineering (10%). Several fields were highly related to the cooking oil remediation study, while some may be less related to the study.

#### 3.4.1. Environmental Science

This field conducts research to identify, control, or eliminate the sources of pollutants or hazards affecting the environment or public health [26]. Environmental research also makes strategies to avoid, control or fix environmental problems such as pollution.

#### 3.4.2. Agricultural and Biological Sciences

This field is related to the soil issues of agricultural land, where remediation of soil improves the quality of cultivated soil and healthy human living environment [27]. In addition, many chemical pollutants could have undesirable effects on nearby water bodies of agricultural land. Hence, bioremediation techniques are developed to solve the problems in the agricultural area.

#### 3.4.3. Chemistry

Bioremediation processes could involve redox reactions, which could trigger chemical reactions on donating and accepting electrons. The specific chemical reactants and products can be determined from the chemical equations for the reactions catalysed by the microbes [28]. Also, soil remediation by chemical treatment encompasses technologies that could destroy or chemically transform the organic matter [18].

#### 3.4.4. Biochemistry, Genetics and Molecular Biology

The reaction of breaking down complex molecules (pollutants) by microorganisms is related to the enzymatic reaction. The microorganisms’ ability to degrade the pollutants depends on the bacteria’s capability to use their functional enzymes to break down the pollutant molecules. For example, lipase-producing bacteria could effectively degrade oil and fats [29]. Moreover, unknown bacteria-degrading pollutants isolated from certain areas (e.g., from contaminated sites) can be identified and further studied by molecular biology [9].

#### 3.4.5. Chemical Engineering

Chemical engineering in the environmental field also examines the assessment of biodegradation methods (biostimulation, bioaugmentation), factors affecting microbial activity and bioavailability of pollutants. Moreover, bioremediation involves using plants or microorganisms, viable or not, natural or genetically engineered to treat environments contaminated. Genetic engineering may improve bioremediation through the engineering of bacteria [30].

### 3.5. Keywords Clustering

**Figure 5:**
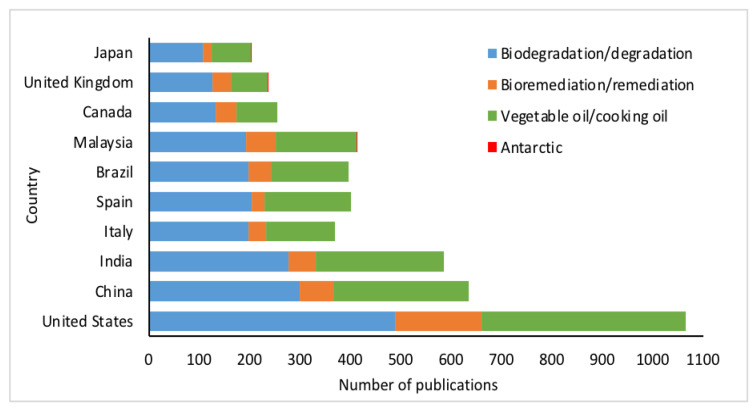
Number of publications based on authors’ keywords according to the top 10 most productive countries.

Keywords are important components in a published research. The purpose of keywords in a research paper is to help researchers in finding the desired articles when searching for a topic. As mentioned earlier, the keywords used in this study were “bioremediation/remediation”, “biodegradation/degradation”, “vegetable oil/cooking oil” and “Antarctic/Antarctica”. These selected keywords were further analysed on the total publications every year for the top 10 countries that have the most publications in this study (refer to Section 3.2). The frequency of the words “biodegradation/degradation” and “bioremediation/remediation” in the United States, India and China as author’s keywords was almost the same, while most of the other countries preferred to use “biodegradation/degradation” as their keywords in this field of studies (Figure 5). At the same time, the United States also has the highest research on vegetable oil and cooking oil remediation, followed by China and Malaysia. The least used keyword in this study was “Antarctic”, with Malaysia (2 publications), the United Kingdom (1 publication) and Japan (1 publication).

Interest in Antarctic research has been developing for almost three decades in many fields including geosciences, ecology and atmospheric sciences [31]. Research on Antarctica has been published from all over the world. The percentage of international articles increased two-fold between 1993 to 2012. A bibliometric analysis on Antarctic research for 1998 to 2015 by Jang et al. (2020) reported that the degree of centrality on the Earth and related environmental sciences were ranked for several countries including the United States America, Germany, France and the United Kingdom [32]. Studies on environmental science started to gain popularity during the emergence of global issues on climate change. The consensus among the scientific community is that this issue is of great concern since Antarctica is the most sensitive continent and originally one of the most undisturbed places on Earth [33].

The Malaysian interest in polar research started in 1983, which was then followed by Malaysia joining as a member of the Antarctica Treaty in 2011. The scientific collaboration has strengthened Malaysia’s interest in promoting Antarctica to preserve its pristine environment [34]. Various studies on environmental science were conducted to clean the oil spills and mostly focusing on diesel degradation treatment in the Antarctic through biological or physiochemical treatment in the past three decades [35,36,37,38]. However, research on the treatment of waste cooking oil in Antarctica (especially because of the challenging cold climate) was non-existent until 2019. Although there was no major cooking oil pollution that happened in Antarctica, there is possibility that it can occur while loading and unloading the oil from supply ships. Thus, Malaysia was the first country to study the bioremediation of cooking oil using biological approaches in Antarctica [29]. The idea for the process of removal waste cooking oil in the Antarctic via in situ bioremediation should be considered, instead of carrying the waste back to the original countries for disposal.

**Figure 6:**
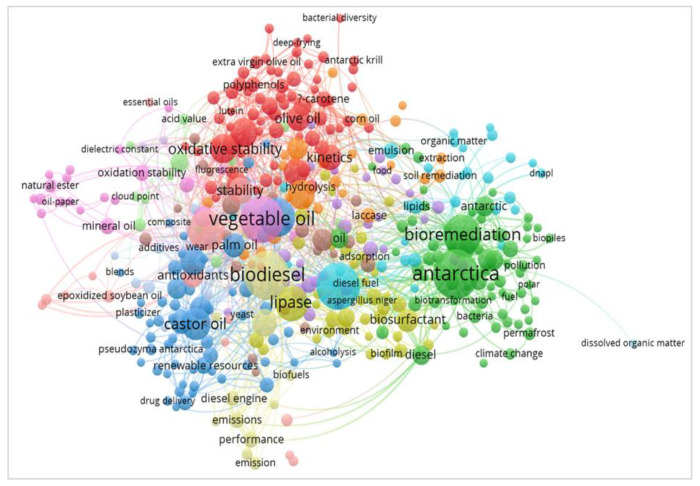
Bibliometric map based on total co-occurrence analysis with network visualization.

The selected keywords were also analysed using VOSviewer software to study the authors’ keywords’ co-occurrence. Figure 6 shows the keywords’ co-occurrences, dispersion and intersection through a bibliometric map with network visualisation. The analysis found 424 intersections of the occurrence and 4470 links in total. The links were designated from the line between the keywords, where the links for biodegradation, bioremediation, remediation, degradation, vegetable oil, cooking oil and Antarctic, Antarctica were 134, 97, 32, 116, 227, 51, 29 and 95, respectively. The bigger the circles in Figure 6, the more the number of occurrences of the keyword.

**Figure 7:**
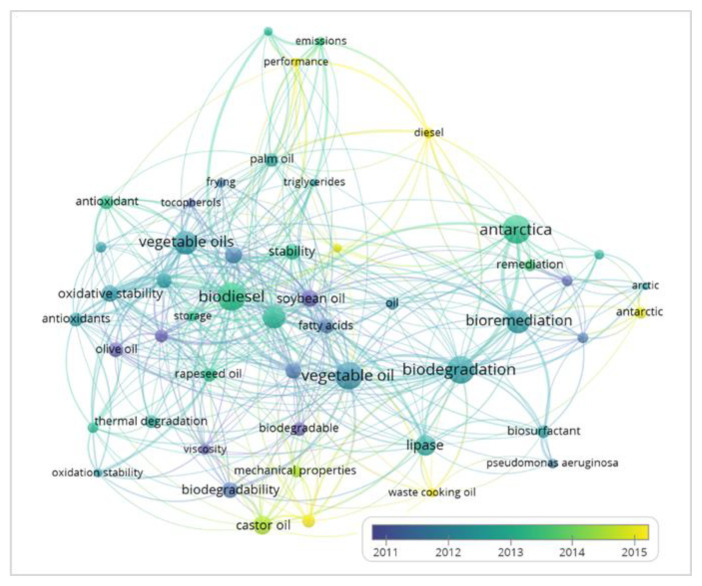
Bibliometric map based on top 50 co-occurrence of keywords analysis with overlay visualization for 2011 to 2015.

The keywords were narrowed down to the top 50 for further analysis using overlay visualisation for 2011–2015. The years were selected because this range was the most significant compared to other years for the keywords (Figure 7). The colour changes indicate the trend in trending topics in this field. The dark blue represents the keywords released before 2012, including “biodegradation”, “bioremediation” and “vegetable oils”. Meanwhile, the trending topics in 2015 were “Antarctic”, “castor oil”, “diesel” and “waste cooking oil”. Hence, the researchers have been paying more attention to the bioremediation of hydrocarbons (diesel and cooking oil) in the Antarctic after 2015.

Amongst all of these keywords, the number of connections to “biodegradation” (38 links) was the highest. This shows their relevance, and these keywords usually appear together in the same research with most researchers focusing on these issues. As mentioned before, lines represent the co-occurrence links between two keywords. The thicker the line between two keywords, the more frequently they appear. The link between biodegradation, bioremediation, vegetable oil and Antarctica showed a thicker line than others. Besides, the link of biodegradation with lipase for biodegradation of waste cooking oil became thicker over a time.

#### 3.5.1. Biodegradation/Bioremediation

The biodegradation keywords were also linked to bioremediation, biosurfactant and vegetable oil (Figure 7), as these keywords are related in this research area. Generally, the biodegradation process involves the breakdown of hydrocarbons allowing the production of biosurfactant compounds from the microorganisms. Biosurfactants are chemical compounds consisting hydrophilic and lipophilic properties that are produced on bacterial cell surfaces or extracellularly excreted [39]. The hydrophilic part (polar group) is responsible for water solubility of the surfactants. In contrast, the hydrophobic portion (non-polar chain) appears to concentrate at air-water interfaces or the micelles’ centre, decreasing the solution’s surface tension [40,41]. Surfactants form micelles, which confers several surfactants’ characteristics, such as emulsifying, foaming, dispersing, and allowing very flexible chemical compounds for surfactants. Most of the bacteria are able to produce biosurfactant compounds during the process of biodegradation or bioremediation of hydrocarbons including diesel, cooking oil, lubricating oil and petroleum [42]. This is because, the biosurfactant compound can increase the surface area of the hydrocarbons, thus allowing more effective in the hydrocarbon degradation process. According to Zakaria et al. (2019), there are various types of biosurfactants produced by bacteria producing Antarctic bacteria with different types of substrates and hydrocarbons [43]. Numerous studies on the ability of various strains of Pseudomonas aeruginosa to produce biosurfactants have been reported over the last three decades [44,45,46,47]. Rhl quorum sensing systems such as RhlA, B, R and I genes are important for *P. aeruginosa* in the production of glycolipid biosurfactants [48]. Rhamnolipids are glycolipid biosurfactants abundantly produced by *P. aeruginosa* including 3-(3-hydroxyalkanoyloxy)alcanoic acid, L-rhamnosyl-3-hydroxydecanoyl-3-hydroxy- decanoate, and L-rhamnosyl-L-rhamnosyl-3-hydroxydecanoyl-3-hydroxydecanoate [49,50]. The ability of rhamnolipids produced by bacteria to efficiently emulsify hydrocarbons has been proven by previous studies through emulsification activity analysis [51,52]. It has also been found that these microbial biosurfactants’ mechanism of action depends on cell surface hydrophobicity, where the cell surface was characterised by low hydrophobicity and reduce the cell surface with high hydrophobicity [53]. Most importantly, biosurfactants are degradable and non-toxic to the environment [54]. This shows that biosurfactants could play a big role in the bioremediation process, especially in cleaning up hydrocarbons pollution.

#### 3.5.2. Antarctica

Antarctica was one of the keywords highly connected to the bioremediation. The remediation field is widely studied by researchers who focused both on the Antarctic and the Artic. As shown in Figure 7, “diesel” appears to be interconnected to “Antarctica”. Initially, during the 1980s, there were many diesel oil spills in Antarctica due to incidents involving vessels during transportation. A huge amount of diesel has been spilled into the seawater and it is time consuming and difficult to remove the pollutants in the cold Antarctic environment. On 28 January 1989, about 600,000 L of diesel have been spilled into the seawater on the Antarctic Peninsula Southern Ocean, which caused the biggest oil spill in the Antarctic ever recorded. Also, about 270,000 litres of light marine diesel have been released into near-shore waters of Macquarie Island during 1987, which affected all the aquatic invertebrates and marine algae. Subsequent incidents and accidents then continued (1997, 2001, 2007, 2009, 2010, 2011) for a decade [55]. This situation attracted researchers’ attention to remediate the diesel pollution in Antarctica. Other than diesel, the keyword “oil” also appeared in the bibliometric map (Figure 7) and it too is linked to “Antarctica”. Other than diesel, natural oils including vegetable oil are widely used for kitchen activities in Antarctica as well as other types of oil such as waste petrol, engine oil and lubricant oil.

#### 3.5.3. Vegetable Oil/Vegetable Oils

The biodegradation of vegetable oil begins with the breakdown of the complex molecules to acetyl compounds by enzymes produced by microbes with various types of lipolytic enzymes through enzymatic hydrolysis [56]. Lipolytic enzymes including lipases (EC 3.1.1.3) and carboxylesterases (EC 3.1.1.1) comprise eight family groups of enzymes. Generally, lipases can hydrolyse water insoluble-substrates such as vegetable oil and carboxylesterases that usually act on water-soluble esters. Lipase exhibits low action at low substrate concentration, but this enzyme’s activity rapidly increases with the concentration of the substrate [57]. Lipase modifies oil through hydrolysis reaction aided by the presence of water during the hydrolysis reactions process. Then, it produces fatty acids and other reactions where alcohol is displaced through a transesterification reaction to produce glycerol [58]. This shows that lipase are the key enzymes for the biodegradation of vegetable oil and that these enzymes could also degrade waste cooking oil since they still contain short chained fatty acids molecules.

#### 3.5.4. Biodiesel

Besides than selected keywords, “biodiesel” appeared to have a strong link with the other keywords. Biodiesel (fatty acids methyl esters) has become one of the fuel sources that could serve as a renewable energy source. The production of biodiesel fuel can be done with the addition of alcohol, in the presence of catalysts through transesterification process [59,60]. High yield in biodiesel production was summarised by Mahlia et al. [61], where most researchers used different type of cooking oil including rapeseed, soybean and palm oil as their starting materials and methanol or ethanol as for the alcohol. Biodiesel can only be produced from cooking oil (vegetable oil, animal fats) because the triglycerides (fatty acids and glycerol) are the primary raw materials for biodiesel production. This can be seen in Figure 7 where the “biodiesel” keyword was connected to various types of cooking oil, including rapeseed oil, olive oil, palm oil and soybean oil. The keyword “vegetable oil” was also found to be near to the “biodiesel” keyword with a high number of occurrences. Hence, “biodiesel” could be also displayed on the bibliometric map since it is related to the selected keywords, especially “vegetable oil”.

## 4. Conclusions

The study on the remediation of cooking oil has seen a generally increasing trend. This research topic can be considered as a developing research topic due to the fluctuating trend seen for most countries in the number of publications. Hence, this research topic can be categorised as a trending topic in the present day. However, the main findings in this study on the research trends of biodegradation of cooking oil specifically in Antarctica cannot be summarised. This is because, the study on biodegradation of cooking oil in Antarctic still developing with the 1 publication on 2018 and 4 publications on 2020.

Overall, the country that had the most publications in this research topic was the United States, with 13.67% of the total for the past 20 years. However, the United States’ publications were higher in 2001 to 2010 but since then, China has gradually increased their output of publications. Meanwhile, *The Journal of Agricultural and Food Chemistry* was chosen by most researchers in this field for publication since the journal also has a high impact factor and is categorised as Q1. In the keyword clustering analysis, “Antarctica”, “biodegradation” and “vegetable oil” were shown as the trending topics in this field. The filed that contributed the most was on the environmental sciences, with 14% of publications coming from this field. Further analysis on the co-occurrence of the keywords found that “biodegradation” has the highest links among all other keywords, with 38 links followed by “vegetable oil” (33 links), which indicated that biodegradation is the main core issue in this research topic. Here, bibliometric analysis can help researchers to understanding the worldwide trend and serves as a guideline for future research.

The study on bioremediation or degradation of cooking oil in Antarctica as one of the environmental microbiology in research scope could be connected to the interdisciplinary area of environmental health sciences and public health. The cooking oil pollution may cause various adverse health outcomes including all organisms in the world through the toxic pollution effects, which can also cause serious environmental health problems. After all, the study on the biodegradation of cooking oil in Antarctica is worth to explore. The Antarctic pollution could affect the Earth’s climate and ocean systems, since there is a connection between this continent and the rest of the world through atmospheric and oceanic circulations. As most of Antarctic’s researchers claim, saving Antarctica will save the world.

## Figures and Tables

**Table 1:** Top 20 of productive journals in the vegetable remediation field.

Journal	Total Number of Publications	Impact Factor	Quartile (2019)	Total Co-Citation
*Journal of Agricultural and Food Chemistry*	85	4.192	Q1	874
*Bioresource Technology*	76	7.539	Q1	1031
*Industrial Crops and Products*	70	4.244	Q1	125
*Journal of the American Oil Chemists Society*	67	1.659	Q3	1232
*Food Chemistry*	56	6.306	Q1	658
*Science of the Total Environment*	40	6.551	Q1	303
*Chemosphere*	34	5.778	Q1	1236
*Environmental Science and Technology*	33	7.864	Q1	1215
*Journal of Hazardous Materials*	33	9.038	Q1	333
*Applied Microbiology and Biotechnology*	30	3.53	Q2	409
*Polymer Degradation and Stability*	26	4.032	Q1	267
*International Biodeterioration and Biodegradation*	25	4.074	Q1	87
*Polar Biology*	25	1.728	Q3	223
*Environmental Science and Pollution Research*	24	3.056	Q2	48
*European Journal of Lipid Science and Technology*	23	2.056	Q3	325
*Journal of Applied Polymer Science*	23	2.52	Q2	472
*Journal of Food Science*	22	2.479	Q2	181
*Cold Regions Science and Technology*	20	2.739	Q1	96
*Journal of Environmental Management*	19	5.547	Q1	43
*Journal of Polymers and the Environment*	19	2.572	Q3	84

## Data Availability

Not applicable.
